# Depressive Symptoms and the Link with Academic Performance among Rural Taiwanese Children

**DOI:** 10.3390/ijerph17082778

**Published:** 2020-04-17

**Authors:** Yujuan Gao, Derek Hu, Evan Peng, Cody Abbey, Yue Ma, Chyi-In Wu, Chia-Yuan Chang, Wei-Ting Hung, Scott Rozelle

**Affiliations:** 1 Rural Education Action Program, Freeman Spogli Institute for International Studies, Stanford University, Palo Alto, CA 94305, USA; yujuan.gao@ufl.edu (Y.G.); dhu21@groton.org (D.H.); pengevan@stanford.edu (E.P.); cjabbey88@gmail.com (C.A.); rozelle@stanford.edu (S.R.); 2 Groton School, Groton, MA 01450, USA; 3 Institute of Sociology, Academia Sinica, Taipei 11529, Taiwan; sss1ciw@gate.sinica.edu.tw (C.-I.W.); chiayuanchang.tw@gmail.com (C.-Y.C.); tommytype21@gmail.com (W.-T.H.)

**Keywords:** depressive symptoms, academic performance, rural Taiwanese children, Xinzhumin

## Abstract

Previous studies reflect a high prevalence of depressive symptoms among Taiwanese adolescents (ages 13–18), but there is an absence of literature related to the risk of depression of children in Taiwan (ages 6–12), particularly among potentially vulnerable subgroups. To provide insight into the distribution of depressive symptoms among children in rural Taiwan and measure the correlation between academic performance, we conducted a survey of 1655 randomly selected fourth and fifth-grade students at 92 sample schools in four relatively low-income counties or municipalities. Using the Center for Epidemiological Studies-Depression Scale (CES-D) we assessed the prevalence of depressive symptoms in this sample, in addition to collecting other data, such as performance on a standardized math test as well as information on a number of individual and household characteristics. We demonstrate that the share of children with clinically significant symptoms is high: 38% of the students were at risk of general depression (depression score ≥ 16) and 8% of the students were at risk of major depression (depression score > 28). The results of the multivariate regression and heterogeneous analysis suggest that poor academic performance is closely associated with a high prevalence of depressive symptoms. Among low-performing students, certain groups were disproportionately affected, including girls and students whose parents have migrated away for work. Results also suggest that, overall, students who had a parent who was an immigrant from another country were at greater risk of depression. These findings highlight the need for greater resource allocation toward mental health services for elementary school students in rural Taiwan, particularly for at-risk groups.

## 1. Introduction

Depression is growing increasingly prevalent among children and adolescents [1]. Worldwide, the risk of depression among youth ranges between 5–20% [2]. A number of studies show that certain subgroups may be more vulnerable to depression, such as low-income youth [3,4]. When children (defined in the present study as youth aged 6–12) and adolescents (defined as youth aged 13–18) suffer from depression, their symptoms are often more serious and their episode durations are frequently longer than adults who suffer from it [5,6]. As depression is linked to a whole host of social, behavioral, and health problems, it is important to identify factors associated with depression among youth as well as vulnerable subgroups early on and intervene promptly [6,7,8,9,10].

One risk factor for depression among children and adolescents frequently cited in the literature is poor academic performance, which studies generally show is associated with risk of depression and as being a predictive factor of future depression [11,12,13,14]. One possible reason academic performance causes depression is heightened stress, as academic stress has been found to lead to depression [15]. Another possible reason is related to the social feedback that results from one’s performance in school [12].

In Taiwan’s education system, in which intensive cram schooling, competitive entrance examinations, and pressure from parents are the norm, children may be particularly vulnerable to mental health problems related to academic performance [16,17,18,19]. Academic stress often caused by school examinations, schoolwork, and homework has been found to be significantly correlated with adolescent depression in East Asian countries [15,20]. Accordingly, a range of studies have demonstrated relatively high rates of major depression among Taiwanese adolescents. For example, one study using the Center for Epidemiological Studies-Depression Scale (CES-D) and a cut-off score of 29 found that 12.3% of the 9586 secondary school students surveyed in urban and rural parts of southern Taiwan were at risk for major depression [21]. Another study of 734 adolescents enrolled in vocational schools in a southern Taiwan city using a cut-off score of 16 discovered that 70% of students were at risk for depression in general [22]. These rates of the risk of depressive symptoms are significantly higher than those observed in western countries using the same scale and cut-off scores among similarly-aged youth [23,24].

In line with the international literature, previous studies conducted in Taiwan indicate that poor academic performance is a risk factor for depression although there is no consensus about the direction of the correlation. Several studies performed in urban areas of Taiwan in China find that academic performance and depression are negatively correlated [25,26]. In contrast, several other research teams found positive correlations [27,28] or no correlation at all [21].

Future efforts to combat depression among youth in Taiwan also would benefit from a better understanding of which sub-populations are more or less vulnerable to developing depression. Similar to studies conducted in other countries, most studies on Taiwanese youth seem to agree that girls are more at risk of developing depression than boys [18,21,29,30]. However, there is less of a consensus regarding the association between being low income and having depression [18,21,30,31].

There are also a number of potentially vulnerable subgroups of Taiwanese youth that have been largely ignored in past research. One subgroup whose mental health is yet unexamined is the children of recent immigrants to Taiwan, or Xinzhumin in Mandarin Chinese. Xinzhumin children, who make up approximately 10% of the population in Taiwanese primary schools (and a much higher share in low-income, rural schools), may face social stigma, have difficulty assimilating into local Taiwanese society, and perform worse academically than their peers [32,33,34,35]. Such children may be potentially vulnerable because studies have shown that youth with lower levels of parental care or engagement are more likely to be depressed [36,37]. In addition, most studies in the Taiwan literature focus primarily on exploring depression among adolescents and not children (i.e., elementary school students in grades 1–6). To our knowledge, the only other study conducted in the past decade that focused on the link between risk of depression and academic performance had a relatively small sample size (s < 700), was exclusively conducted in urban schools, and did not report the overall share of high-risk students in the sample [25]. This is problematic, as a number of studies in other East Asian countries, like mainland China and South Korea, have shown that depression rates of elementary school students can be similar to those among older youth [38,39].

In order to fill these gaps in the existing literature, we seek to document the prevalence of depression among rural Taiwanese elementary students and explore the links of depression with academic performance. To meet this overall goal, this study has three specific objectives. First, we seek to document the overall rate of depression among rural Taiwanese elementary students. Second, we measure the correlation between academic performance and rate of depression in the overall sample. Finally, we will explore whether or not the link between academic performance and the rate of depression is stronger among certain subgroups—including girls, Xinzhumin, and children whose parents migrated away for work—by performing heterogeneous analysis, looking specifically at a number of student and family characteristics. Ultimately, we hope our work will be able to raise awareness about certain vulnerable subgroups of rural children to help better target policies to improve their mental health.

In this study, we choose to focus on rural children because previous research has shown that adolescents in rural Taiwan are both outperformed academically by and are more likely to suffer from depression than their urban peers [40,41]. No research study as of yet, however, has specifically investigated the link between academic performance and depression among primary school students in rural areas. Additionally, Xinzhumin—one of our main subgroups of interest in this study—make up a relatively higher proportion of the student population in rural areas compared with urban areas [32].

## 2. Materials and Methods 

### 2.1. Ethical Approval

Ethical approval for this study was granted by the Stanford University Institutional Review Board (IRB) (Protocol ID 35635). All subjects gave written informed consent in accordance with the Declaration of Helsinki. 

### 2.2. Sampling Selection

The data for the present study were collected in the fall of 2018 from four different counties/municipalities which have relatively low levels of economic development in Taiwan: Miaoli county, Yunlin county, Jiayi county, and Tainan. As county-level data for GDP were not available, we use primary income as a measure of economic development, which corresponds to the income of households as a result of participation in the production process. Miaoli and Yunlin are located in central Taiwan and rank 11th and 12th, respectively, out of 20 counties and municipalities. Jiayi is a county in southern Taiwan with a primary income rank of 15. Tainan, a special municipality ranked 6th overall, has the lowest primary income of any special municipality in southern Taiwan [42]. According to official statistics from Taiwan’s Ministry of Education, the three counties chosen for this study also have among the highest proportions of Xinzhumin that make up the local population relative to the rest of Taiwan, while Tainan has among the highest number of Xinzhumin overall [32].

The sampling strategy for our survey was as follows. First, we obtained a list of all rural elementary schools from the local bureaus of education of each county or special municipality. After excluding schools that were unwilling to participate, the research team included 92 schools in the study. Next, in each sample school, we randomly selected one class in fourth grade and one class in fifth grade, enrolling all students in the sample classes into our study. In total, 1655 fourth and fifth-grade students participated.

### 2.3. Data Collection

During November and December of 2018, near the end of the first semester of the 2018–2019 academic year, a three-part survey was conducted by an enumeration team made up of local university students. Enumerators received several days of training from the survey leaders at Academia Sinica before visiting the 92 sample schools. At each sample school, enumerators followed a strict protocol when administering each part of the survey, including providing the sample classes a detailed explanation of the survey expectations and enforcing strict time limits.

In the first part of the survey, we measured depressive symptoms using the Center for Epidemiologic Studies Depression Scale (CES-D). The CES-D was created in the US for measuring the risk of depression and depressive symptoms in 1977 [43]. Since then it has become one of the most widely used assessments of depressive symptoms around the world, and the literature has confirmed its appropriateness for assessing the risk of depression among children and adolescents [44,45]. The reliability and validity of the test, which was translated into Chinese in 1985, has been validated among Taiwanese populations [46,47,48,49,50]. For example, in terms of overall validity, a study about adolescents in Taipei found the CES-D had moderate (meaning an AUC of 0.7–0.9) to high (meaning an AUC of > 0.9) test accuracy for measuring major depressive disorder [50]. Additionally, in our study the internal consistency of reliability of the CES-D Scale, measured by Cronbach’s alpha, is 0.83, which is indicative of a high degree of reliability. Due to its relatively high reliability and validity, the CES-D has been used widely in studies in Taiwan to evaluate symptoms of depression [21,22,49,50,51,52].

The self-report assessment includes 20 items, and has four separate factors: “lack of positive effect”, “depressed affect”, “somatic symptoms”, and “interpersonal problems” [43]. An example of one of these 20 items is: “I was bothered by things that usually don’t bother me”. CES-D is scored on a Likert scale with four possible answers corresponding to how often the respondents experienced a given emotion or performed a certain action within the past week: “rarely or none of the time” (less than 1 day), “some or a little of the time” (1–2 days), “occasionally or a moderate amount of time” (3–4 days), and “most or all of the time” (5–7 days). These answers are scored as 0, 1, 2, or 3, respectively. CES-D scores range from 0 to 60 and a higher score indicates the presence of more symptomatology. Different factors include different numbers of items and therefore have different score ranges: the “lack of positive effect” factor includes four items and scores range between 0 to 12; the “depressed affect” factor includes eight items and scores range between 0 to 24; the “somatic symptoms” factor includes six items and scores range between 0 to 18; and the “interpersonal problems” factor includes two items and scores range between 0 to 6 [46]. 

In addition to calculating the mean score of our sample and sub-groups, we also referred to commonly-used cut-off scores to determine the risk for different severity levels of depression [21,43]. Following the literature, in the present study a score of 16 or higher indicates that a student is at risk of “general depression” (defined as any type of mild, moderate, or major depression) and a score of 29 or higher indicates that a child is at risk of “major depression.”

The second part of the survey involved a standardized math test. The tests (one for fourth grade and one for fifth grade) were jointly designed by Taiwan Academia Sinica and local primary school teachers, and they were fully based on Taiwan’s primary school math curriculum. None of the teachers whose students took part in the survey, however, were involved in the test design, thereby eliminating the risk that certain teachers would prepare their teachers for the test beforehand. The students were required to finish the math tests in 30 min, and the enumeration team closely proctored exams in order to strictly enforce time limits and minimize cheating. For the analysis, the scores were standardized by scaling them into z-scores, which was done by subtracting the mean score and dividing by the standard deviation (SD) of the math score distribution of all students in each grade. These standardized math test scores are used as a measure of academic performance.

The third part of the survey was administered by asking students to fill out a questionnaire that collected data on each student’s individual and family characteristics. The survey form asked about each student’s gender, age, whether or not they are Xinzhumin, family structure, and their parent’s education status. The questionnaire also asked students about whether or not their parents lived with them during most of the last semester. Finally, in accordance with the principal component analysis approach to assess household income, we asked participants whether or not their households had the following list of common items in order to construct an asset-based wealth index: tap water, toilet, water heater, washing machine, computer, internet, refrigerator, air conditioner, motor or electric bicycle, and car [53].

### 2.4. Statistical Analysis

We first examined the prevalence of depression overall and across subgroups. In order to understand which subgroups of students are more likely to be depressed, we compared the subgroups across three measures: the share of the subgroup risk of general depression, the share of the subgroup at risk of major depression, and the average depression score of the subgroup. The subgroups (which are student and family characteristics) that we include are student gender, age, whether the student has a parent who is an immigrant (Xinzhumin), academic performance, whether the student is an only child, the education level of the parents, whether the parent lived at home last semester, and household consumption asset value. We used t-tests to measure if there is a significant difference between those groups and analyzed which characteristics are correlated with depression.

Next, in estimating the correlation between depression and academic performance, we ran multivariate analysis with the addition of a vector of control variables. The function is below:

(1) $$y_{i}=\alpha +\beta_{1}score_{i}+\gamma x_{i}+\Phi c_{\;}+\varepsilon_{i}$$

where the dependent variable $y_{i}$ indicates the depression level of student *i*. This has several different measures, including whether the student is at risk of general depression (equals 1 if score ≥ 16), whether the student is at risk of major depression” (equals 1 if score > 28), and the student total depression score as well as depression score of each factor. Score indicates the standardized mathematics score.

The vector $x_{i}$ includes student individual and family characteristics. The student individual characteristics include student gender (equals 1 if the student is male), student age (in years), as well as whether the student is an only child (equals 1 if the student is an only child) and whether the student is Xinzhumin (equals 1 if the student is Xinzhumin). The family characteristics include the education level of parents (equals 1 if the father/mother of the student has graduated from high school), parental migrant status (equals 1 if the father/mother of the student did not live with children during the most of last semester), and household consumption asset value (equals 1 if the household is in the lower 50 percentile of the sample). To further improve statistical efficiency, we add county-level fixed effects (represented by wj) and compute robust standard errors (adjusted for clustering at the school level).

We also performed heterogeneous analysis to observe whether or not there was interaction between certain variables of interest (including gender, Xinzhumin status, and parental migration status) and mathematics score on depressive symptoms. Female students have been previously identified as a high-risk subgroup in the international and Taiwanese literature. Xinzhumin are also a potentially vulnerable group due to their relatively poorer academic performance historically and the social stigma they often face [21,54]. Additionally, previous studies have found that left-behind children have worse academic performance than their peers. To perform this heterogeneous analysis, we added interaction terms between the math score and variable of interest into the basic model.

## 3. Results

### 3.1. Descriptive Statistics

The descriptive statistics of our sample are displayed in Table 1. As can be seen from the data, the mean age for all the students in our sample was 10.41 years old (ranging between 10 to 15 years old). In terms of individual characteristics, 52% (867/1655) of students were male, 11% (187/1655) were the only child in their family, and 22% (363/1655) were Xinzhumin. 

For the household characteristics, 49% (805/1655) of the students had fathers who completed high school and 51% (844/1655) had mothers who completed high school. The parents of the minority of students migrated out: 14% (227/1655) had fathers who did not live at home during most of the previous semester, and 13% (223/1655) had mothers who did not live at home during most of the previous semester. 

### 3.2. Prevalence of Depression among the Sample

Table 2 summarizes the depressive symptoms in our sample, categorized by CES-D scores. As can be seen in the table, the overall mean depression score for the students in our sample was 14.60 points. In terms of different factors, the mean score was 4.25 points for the “lack of positive affect” factor, 5.02 points for the “depressed affect” factor, 4.19 points for the “somatic symptoms” factor, and 1.14 points for the “interpersonal problems” factor. In terms of different levels of severity, 38% of the students were at risk of having general depression (depression score ≥ 16), and 8% of students were at risk of having major depression (depression score > 28).

### 3.3. Correlation between Depressive Symptoms and Academic Performance 

First, according to the results of Table 3 and Table 4, we observed a significant correlation between depressive symptoms and a certain number of student and household characteristics. Specifically, those subgroups that exhibited at least one depressive factor include younger students, boys, Xinzhumin students, students with fathers who completed high school, students with migrant parents, and students from poorer households (Table 3, rows 2–10, columns 1–4). Besides male students, the above subgroups also were at greater risk to suffer from depression (either general or major—Table 4, rows 2–10, columns 1–2).

After controlling for student, family, and school characteristics (with fixed effects), we found that students with higher math scores have significantly fewer depressive symptoms (Table 3). Specifically, a one-standard deviation increase in a student’s math score is associated with a 1.90-point decrease in the depression score (row 1, column 1, significant at the 1% level). In terms of different depression factors, a one standard deviation increase in the mathematics score is associated with a 0.48-point decrease in the “lacking of positive affect” factor, a 0.66-point decrease in the “depressed affect” factor, a 0.56-point decrease in the “somatic symptoms” factor, and a 0.20-point decrease in the “interpersonal problems” factor. These associations are all significant at the 1% level (row 1, columns 2–4).

We continue to find consistent results in terms of the correlation between academic performance and the risk of depression: students with higher math scores were significantly less likely to be at risk for depression (Table 4). Higher math scores translated to a 9-percentage point decrease in the risk of general depression and a 3-percentage point decrease in the risk of major depression (row 1, column 1–2, significant at the 1% level).

### 3.4. Heterogeneous Analysis 

Table 5 and Table 6 demonstrate how the association between academic performance and risk for depression differ among certain subgroups after adjusting for potential confounders. 

#### 3.4.1. Gender and Ethnicity

According to Table 5, Panel A, academic performance and risk for depression are negatively correlated for both genders, though girls who struggle academically are significantly more likely to be at risk for major depression than male classmates who perform similarly poorly. Row 1 columns 1–7 show that girls with poorer academic performance are significantly more likely than academically superior female classmates to be at risk for depression across all factors and severity levels. Row 4 columns 1–7 show similar results for poorly performing male students. Row 3 column 7, however, indicates that there is a difference between the share of poor-performing girls and boys who are at risk of having major depression: girls are significantly more likely than boys to be at risk for major depression if they perform poorly academically (significant at the 10% level). The correlation between academic performance and risk for depression is statistically insignificant for the other measures of risk for depression and depressive symptoms. 

Panel B of the same table shows a different scenario for non-Xinzhumin and Xinzhumin students, which do not exhibit any significant heterogeneous differences in regard to the correlation between the two variables of interest. Overall, both non-Xinzhumin and Xinzhumin students are significantly more likely to exhibit depressive symptoms if they perform poorly at school. In the case of non-Xinzhumin students, this is apparent across all measures of measures of depression (columns 1–7) while for Xinzhumin students this is the case for the majority of the seven measures of depression (columns 1,3,4,6). According to row 7, there is no significant difference in the strength of the correlation between academic performance and risk of depression for any of the seven measures. Thus, while Xinzhumin might overall be more likely to be at risk for developing major depression (row 6 column 7), it does not appear that their risk is more strongly associated with poor performance at school in comparison to that of their peers.

#### 3.4.2. Parental Migration Status

Table 6 compares the correlation of the two variables of interest between students whose parent migrated and those whose parents lived at home for most of the last semester, separately presenting results based on parental gender (Panels A and B). Panel A indicates that both poor-performing children whose fathers migrated and those whose fathers lived at home were more likely than academically superior peers to be at risk for depression across most measures. For one of these measures, the lack of positive affect, there was significant heterogeneity: students whose fathers migrated were significantly more likely to be experience a lack of positive affect than those whose fathers lived at home (row 3 column 2, significant at the 5% level). There were no significant differences according to the other depression measures.

Panel B of Table 6 tells a different story for those students whose mothers migrated away from home, however. Although students who struggled academically were more likely to be at risk for depression regardless of maternal migration status, those students whose mothers did not migrate were at greater risk. Poor-performing students whose mothers were at home were both more likely to experience depressed affect (row 7 column 3, significant at the 10% level) and to be at risk for major depression (row 7 column 7, significant at the 1% level) than those whose mothers migrated. This is in contrast not only to the corresponding results regarding paternal migration (see Panel A and paragraph above) but also to the correlations between maternal migration and most measures of depression (Panel B, row 6), which are negative and significant. In other words, although mothers’ presence at home was associated with a lower risk for their children’s depression in general, children who performed poorly at school were more likely to be at risk for depression if their mother lived at home.

## 4. Discussion

In this paper we explored the prevalence of depressive symptoms and its correlation with academic performance across different subgroups of elementary school students in rural Taiwan. Our data, based on responses from 1655 fourth and fifth graders, demonstrate that overall 38% of the students were at risk of general depression (depression score ≥ 16) and 8% of the students were at risk of major depression (depression score>28), with an average sample depression score of 14.60 points. 

Comparisons with other populations of children and adolescents where the CES-D has also been used with the same cut-off scores reveal that depressive symptoms are highly prevalent in rural Taiwanese elementary schools. First, when we compare these rates of depression risk to those of similarly-aged (and even poorer) rural children in an East Asian neighbor with a notoriously high-pressure schooling system, we find that they are even higher or at least comparable. Zhou et al. [55] observed that among 1990 rural children aged 10 to 15 years old in mainland China, 23% were at risk of general depression (versus 38% in our sample) and only 2% were at risk of major depression (versus 8% in our sample). Another study conducted in rural China using the same cut-off score for general depression [56] found that 42% of the 8 to 14-year-old children were at risk (4 percentage points higher than in our sample). It is important to note, however, that the sample in that study was slightly older and was predominantly made up of a particularly vulnerable subgroup of rural Chinese children who live without their parents (893 out of 1228 children).

Second, when we compare these rates and this mean score to those of adolescents in western countries, it is clear that depressive symptoms are more prevalent in rural Taiwan despite our younger sample. Olsson and Von Knotting [23] found that their sample of 2272 Swedish adolescents aged 16–17 had a mean score of 13.4 (versus 14.60 in our sample), and Bergen et al. [24] reported that a sample of 2603 Australian adolescents (average age 13) had a mean score of 12.4, with 26% of students categorized as at risk of general depression (mean score ≥ 16). These results suggest, then, that in an international context income and age may not matter as much as other factors such as the local schooling and societal environment.

However, when comparing our results with the results of studies conducted on Taiwanese adolescents, it still appears that there is a higher prevalence among older youth in Taiwan compared to younger youth [21,22,49,50]. These findings align with the general consensus in the existing literature, which show that depression becomes more common in the teenage years [57,58]. Besides possible hormonal and developmental explanations for the apparent lower risk of depression among children [59,60], increasing pressures in secondary school to excel academically may also be an important reason, particularly in Taiwan.

After controlling for student and family characteristics, our paper shows that poor academic performance, the main independent variable of interest in this study, was a major risk factor for depression among rural Taiwanese youth. As mentioned in the introduction, this association may be related to the academic stress that is often found in education systems with high-stakes examinations like Taiwan. Greater academic stress has been shown, in turn, to lead to depression [15]. Another—perhaps concurrent—possibility, as discussed by Chen, Rubin and Li [12], is that the origin of the depression is exogenous, with struggling students receiving negative feedback from their social surroundings, thereby exacerbating the symptoms they may already feel.

When comparing the correlation between academic performance and depressive symptoms across different vulnerable populations, the results suggest that certain subgroups in rural Taiwan are significantly more likely to be at risk for depression, especially in the face of poor academic performance. First, although we found that girls are no more likely than boys to develop depression in general, it appears that low-performing female students are at a greater risk for developing depression than low-performing male students. According to a previous study, girls who subjectively viewed their grades as poor were more likely than boys to have depression [14]. This indicates that actions must be taken to prevent girls who struggle in school from developing mental health issues. 

In addition, we found that although Xinzhumin (children who have one or two non-native parents) were more likely to be at risk for major depression than their peers in general, low-performing Xinzhumin were not any more likely than other low-performing students to experience depressive symptoms. It is also relevant to note that there was no significant difference in the academic performance between the Xinzhumin in our sample and their peers (Table A1). Taken together, this suggests that academic performance may not be an area where Xinzhumin disproportionately struggle, which may be at least in part due to the importance placed on work ethic and social advancement by Xinzhumin mothers [61]. This also suggests, however, that there are likely other reasons besides academic performance that can explain their relatively poorer mental health, which warrants further investigation in future studies.

Finally, we discovered that students whose parents migrated away from home were also at greater risk for depression. This finding supports previous research which suggests that decreased parent-child interaction may result in the development of mental health issues [62]. However, our analysis also produced an unexpected result: the association between academic performance and risk for depression depended on which parent migrated. As expected, those students who faced academic difficulties were more likely to be at risk for depression if their fathers migrated than if their fathers lived at home for most of the previous semester. In contrast, however, the opposite was true for maternal migration: poor-performing students were less likely to be at risk for depression if their mothers migrated than if they lived at home. One plausible explanation is that students may receive more authoritative parenting from mothers in Taiwan and thus suffer from greater degrees of academic stress, as mothers have traditionally been more involved in their children’s education while fathers’ traditional duty has been to provide for the household financially [63]. This, however, is only a preliminary hypothesis.

This study has a number of strengths and contributions. First, our study has a large sample size, with a total of 1655 fourth and fifth grade students from 92 rural schools located in four different counties. Second, our sample explores the risk of depression among Xinzhumin children, which are an understudied and yet potentially vulnerable group in Taiwan. Third, ours is perhaps the only study in recent years to explore the association between depression and academic performance among low-income, rural Taiwanese primary school students, as most other studies in Taiwan focus on adolescent urban students.

Even with its strengths, our study also has several limitations. First, as our study was conducted using baseline data, we were only able to identify the correlation between depression and academic performance and could not prove causation. Second, our rural sample was not nationally representative, as we collected the data in counties with relatively low levels of income.

From both a research and policy perspective, our results suggest that the mental health of elementary school students in rural Taiwan cannot be ignored and that resources need to be concentrated on particularly vulnerable subgroups. Despite previous research that suggests that children have a low prevalence of depression, our study indicates that a significant share of pre-pubescent rural Taiwanese children may be at risk of depression. Moreover, our study provides strong evidence that a major risk factor for depression among Taiwanese children is poor academic performance. In order to ensure the healthy development and prosperous future of Taiwan’s youth, efforts need to be taken in both the schools and in the home to provide interventions already shown to effectively prevent the onset of depression among youth (such as cognitive-behavioral training) and emotional support to children struggling academically, especially for struggling female students and left-behind children [64].

## 5. Conclusions

This study makes a valuable contribution by examining the correlation between academic performance and depression in rural Taiwanese primary schools. Our findings suggest that poor academic performance is a major factor for depression and effective interventions are needed to prevent the development of mental health issues among certain disadvantaged groups. In rural Taiwan, academically-struggling girls, academically-struggling children with migrant parents, and Xinzhumin may be particularly at risk.

## Figures and Tables

**Table 1:** Descriptive statistics of student and household characteristics, n = 1655.

No.	Variables	All Sample	
		Mean/Frequency and Percentage	Std. Dev.
1	Student characteristics		
2	Age (years)	10.41	0.76
3	Male, *n* (%)	0.52	0.50
4	Only child, *n* (%)	0.11	0.32
5	Xinzhumin, *n* (%)	0.22	0.41
6	Household characteristics		
7	Father completed high school or above, n (%)	0.49	0.50
8	Mother completed high school or above, n (%)	0.51	0.50
9	Paternal migration (1 = yes), n (%)	0.14	0.34
10	Maternal migration (1 = yes), n (%)	0.13	0.34
11	Asset index (PCA score), n (%)	0.06	1.27

Note: Data source is authors’ survey.

**Table 2:** Depressive symptoms across entire sample, *n* = 1655.

No.	Variables	Mean/Percent	Std. Dev.
1	Depression score *	14.60	8.96
2	Lack of positive affect *	4.25	3.07
3	Depressed affect *	5.02	4.40
4	Somatic symptoms *	4.19	3.29
5	Interpersonal problems *	1.14	1.46
6	General depression (Depression score ≥ 16; 1 = Yes)	0.38	0.48
7	Major depression (Depression score > 28; 1 = Yes)	0.08	0.27

Note: Data source is authors’ survey. * is continuous measurement; the higher score indicates more depressive symptoms.

**Table 3:** **Ordinary least square** (OLS) regression on correlation between depressive symptoms and academic performance across different factors.

No.	Student Characteristics	(1)	(2)	(3)	(4)	(5)
		Depression Score	Lack of Positive Affect	Depressed Affect	Somatic Symptoms	Interpersonal Problems
1	Math score (SD)	−1.90 *** (0.24)	−0.48 *** (0.08)	−0.66 *** (0.11)	−0.56 *** (0.10)	−0.20 *** (0.04)
2	Age (years)	−0.37 (0.29)	0.14 (0.10)	−0.19 (0.14)	−0.26 ** (0.10)	−0.06 (0.05)
3	Male (1 = yes)	0.05 (0.38)	0.06 (0.14)	−0.21 (0.19)	0.28 * (0.14)	−0.07 (0.06)
4	Only child (1 = yes)	−0.32 (0.63)	−0.35 (0.25)	−0.05 (0.31)	0.10 (0.29)	−0.02 (0.12)
5	Xinzhumin (1 = yes)	0.82 (0.53)	0.41 ** (0.19)	0.18 (0.25)	0.21 (0.19)	0.02 (0.10)
6	Father completed high school or above (1 = yes)	0.60 (0.47)	0.06 (0.15)	0.21 (0.24)	0.17 (0.17)	0.16 ** (0.08)
7	Mother completed high school or above (1 = yes)	−0.69 (0.56)	−0.13 (0.21)	−0.36 (0.27)	−0.08 (0.19)	−0.12 (0.08)
8	Paternal migration (1 = yes)	1.34 ** (0.62)	0.27 (0.19)	0.54 * (0.30)	0.38 (0.23)	0.16 (0.13)
9	Maternal migration (1 = yes)	1.35 * (0.70)	0.52 ** (0.21)	0.64 * (0.37)	0.08 (0.30)	0.12 (0.12)
10	Richer household (1 = yes)	−0.69 (0.47)	−0.32 ** (0.14)	−0.04 (0.25)	−0.29 * (0.16)	−0.04 (0.09)
11	County fixed effects	Yes	Yes	Yes	Yes	Yes
12	Constant	21.39 *** (3.07)	3.52 *** (1.15)	8.50 *** (1.62)	7.53 *** (1.06)	1.84 *** (0.59)
13	Observations	1655	1655	1655	1655	1655
14	R-squared	0.07	0.06	0.04	0.05	0.04

Note: Data source is authors’ survey. Robust standard errors in parentheses are clustered at school level; * indicates significant at 10%; ** indicates significant at 5%; *** indicates significant at 1%.

**Table 4:** OLS regression on correlation between depressive symptoms and academic performance across different levels of severity.

No.	Student Characteristics	(1)	(2)
		General Depression (Depression Score ≥ 16, 1 = yes)	Major Depression (Depression Score > 28, 1 = yes)
1	Math score (SD)	−0.09 *** (0.01)	−0.03 *** (0.01)
2	Age (years)	−0.01 (0.02)	−0.02 * (0.01)
3	Male (1 = yes)	−0.01 (0.02)	0.01 (0.01)
4	Only child (1 = yes)	0.02 (0.03)	−0.01 (0.02)
5	Xinzhumin (1 = yes)	0.01 (0.03)	0.04 * (0.02)
6	Father completed high school or above (1 = yes)	0.06 ** (0.02)	0.00 (0.01)
7	Mother completed high school or above (1 = yes)	−0.04 (0.03)	−0.02 (0.02)
8	Paternal migration (1 = yes)	0.04 (0.03)	0.05 * (0.02)
9	Maternal migration (1 = yes)	0.07 * (0.04)	−0.00 (0.02)
10	Richer household (1 = yes)	−0.04 * (0.02)	−0.00 (0.01)
11	County fixed effects	Yes	Yes
12	Constant	0.62 *** (0.17)	0.32 *** (0.11)
13	Observations	1655	1655
14	R-squared	0.05	0.03

Note: Data source is authors’ survey. Robust standard errors in parentheses are clustered at school level; * indicates significant at 10%; ** indicates significant at 5%; *** indicates significant at 1%.

**Table 5:** OLS regression results showing heterogeneous effects of academic performance on depressive symptoms across gender and Xinzhumin status, *N* = 1655.

No.	Student Characteristics	(1)	(2)	(3)	(4)	(5)	(6)	(7)
		Depression Score	Lack of Positive Affect	Depressed Affect	Somatic Symptoms	Interpersonal Problems	General Depression	Major Depression
Gender (Panel A)								
1	Math score (SD) ^a^	−2.11 *** (0.37)	−0.51 *** (0.12)	−0.79 *** (0.17)	−0.61 *** (0.15)	−0.20 *** (0.06)	−0.09 *** (0.02)	−0.05 *** (0.01)
2	Male (1 = yes)	0.06 (0.38)	0.06 (0.14)	−0.21 (0.19)	0.28 * (0.14)	−0.07 (0.06)	−0.01 (0.02)	0.01 (0.01)
3	Male * Math score	0.35 (0.50)	0.05 (0.15)	0.22 (0.24)	0.08 (0.18)	0.01 (0.08)	−0.00 (0.02)	0.03 * (0.01)
4	Male = No.1 + No.3 ^b^	−1.75 *** (0.32)	−0.46 *** (0.10)	−0.57 *** (0.16)	−0.53 *** (0.12)	−0.20 *** (0.06)	−0.09 *** (0.02)	−0.02 * (0.01)
Whether student is Xinzhumin (Panel B)								
5	Math score (SD) ^c^	−2.05 *** (0.26)	−0.53 *** (0.08)	−0.69 *** (0.13)	−0.59 *** (0.10)	−0.23 *** (0.04)	−0.09 *** (0.01)	−0.04 *** (0.01)
6	Xinzhumin (1 = yes)	0.82 (0.53)	0.41 ** (0.18)	0.18 (0.25)	0.21 (0.19)	0.02 (0.09)	0.01 (0.03)	0.04 ** (0.02)
7	Xinzhumin * Math score	0.70 (0.61)	0.26 (0.17)	0.16 (0.30)	0.14 (0.24)	0.14 (0.09)	0.03 (0.03)	0.02 (0.02)
8	Xinzhumin = No.5 + No.7	−1.35 ** (0.56)	−0.28 (0.17)	−0.53 ** (0.26)	−0.45 ** (0.22)	−0.09 (0.09)	−0.06 ** (0.03)	−0.02 (0.02)

Notes: Data source is authors’ survey. All regressions control for student, household characteristics and county fixed effects. Robust standard errors (in parentheses) are clustered at the school level. * indicates significant at 10%; ** indicates significant at 5%; *** indicates significant at 1%. ^a^ Association between math score and depression for female students; ^b^ Association between math score and depression for male students; ^c^ Association between math score and depression for non-Xinzhumin students; d Association between math score and depression for Xinzhumin students.

**Table 6:** OLS regression results showing heterogeneous effects of academic performance on depressive symptoms between different parental migration status, *N* = 1655.

No.	Student Characteristics	(1)	(2)	(3)	(4)	(5)	(6)	(7)
		Depression Score	Lack of Positive Affect	Depressed Affect	Somatic Symptoms	Interpersonal Problems	General Depression	Major Depression
Father migration status (Panel A)								
1	Math score (SD) ^a^	−1.81 *** (0.24)	−0.42 *** (0.09)	−0.63 *** (0.12)	−0.57 *** (0.09)	−0.19 *** (0.04)	−0.09 *** (0.01)	−0.03 *** (0.01)
2	Paternal migration (1 = yes)	−1.35 ** (0.60)	−0.27 (0.19)	−0.54 * (0.30)	−0.38 (0.23)	−0.16 (0.13)	−0.04 (0.03)	−0.05 * (0.02)
3	Paternal migration * Math score	−0.78 (0.77)	−0.48 ** (0.19)	−0.29 (0.36)	0.08 (0.30)	−0.08 (0.13)	−0.00 (0.04)	−0.01 (0.02)
4	Paternal migration = No.1 + No.3 ^b^	−2.59 *** (0.75)	−0.90 *** (0.17)	−0.92 ** (0.35)	−0.49 (0.30)	−0.28 ** (0.12)	−0.09 ** (0.04)	−0.04 * (0.02)
Mother migration status (Panel B)								
5	Math score (SD)	−2.05 *** (0.23)	−0.47 *** (0.08)	−0.75 *** (0.11)	−0.62 *** (0.10)	−0.22 *** (0.04)	−0.09 *** (0.01)	−0.04 *** (0.01)
6	Maternal migration (1 = yes)	−1.44 ** (0.70)	−0.51 ** (0.21)	−0.69 * (0.37)	−0.11 (0.30)	−0.13 (0.12)	−0.07 * (0.04)	−0.00 (0.02)
7	Maternal migration * Math score ^c^	1.08 (0.68)	−0.09 (0.26)	0.64 * (0.33)	0.40 (0.25)	0.12 (0.11)	0.03 (0.03)	0.06 *** (0.02)
8	Maternal migration = No.5 + No.7 ^d^	−0.97 (0.69)	−0.55 ** (0.25)	−0.11 (0.34)	−0.21 (0.23)	−0.10 (0.10)	−0.06 * (0.03)	0.02 (0.02)

Notes: Data source is authors’ survey. All regressions control for student, household characteristics and county fixed effects. Robust standard errors (in parentheses) are clustered at the school level. * indicates significant at 10%; ** indicates significant at 5%; *** indicates significant at 1%. ^a^ Association between math score and depression for students whose father did not migrate last semester; ^b^ Association between math score and depression for students whose father migrated last semester; ^c^ Association between math score and depression for students whose mother did not migrate last semester; ^d^ Association between math score and depression for students whose mother migrated last semester.

## Appendix A

**Table A1:** Difference of academic performance between Xinzhumin and Non-xinzhumin students.

Variables	Observations	Math Score (SD)	
		Mean	Difference (*p*-Value)
Xinzhumin	363	0.00	−0.02
Non-xinzhumin	1292	0.02	(0.71)

Notes: Data source is authors’ survey.
